# The online attention to orthodontic research: an Altmetric analysis of the orthodontic journals indexed in the journal citation reports from 2014 to 2018

**DOI:** 10.1186/s40510-020-00332-6

**Published:** 2020-09-21

**Authors:** Daniele Garcovich, Angel Zhou Wu, Ana-Matilde Sanchez Sucar, Milagros Adobes Martin

**Affiliations:** 1 Department of Orthodontics, European University of Valencia, Valencia, Spain; 2 Department of Paediatric Dentistry, Dental School, University of Valencia, Valencia, Spain

**Keywords:** Altmetrics, Bibliometric, Citations count, Mendeley, Social media

## Abstract

**Background:**

To describe the impact of research, beyond the limits of the academic environment, Altmetric, a new social and traditional media metric was proposed. The aims of this study were to analyze the online activity related to orthodontic research via Altmetric and to assess if a correlation exists among citations, Mendeley reader count, and the AAS (Altmetric Attention Score).

**Method:**

The Dimensions App was searched for articles published in the orthodontic journals listed in the Journal Citation Reports (JCR) throughout the years 2014 to 2018. The articles with a positive AAS were collected and screened for data related to publication and authorship. The articles with an AAS higher than 5 were screened for research topic and study design. Citation counts were harvested from Web of Science (WOS) and Scopus.

**Results:**

The best performing journals were Progress in Orthodontics and the European Journal of Orthodontics with a mean AAS per published item of 1.455 and 1.351, respectively and the most prevalent sources were Tweets and Facebook mentions. The most prevalent topic was Oral Health-Related Quality of Life (OHRQOL) and the study design was systematic reviews. The correlation between the AAS and the citations in both WOS and Scopus was poor (*r* = 0.1463 and *r* = 0.1508, *p* < .05). The correlation between citations count and Mendeley reader (*r* = 0.6879 and *r* = 0.697, *p* < .05) was moderate.

**Conclusions:**

Few journals displayed a high level of web activity. Journals and editors should enhance online dissemination of the scientific outputs. The authors should report the impact of the findings to the general public in a convenient way to facilitate online dissemination but to avoid an opportunistic use of the research outputs. Despite the lack of correlation, a combination of the citation count and the AAS can give a more comprehensive assessment of research impact.

## Background

Citations metrics as the impact factor or the h-factor, among others, have been extensively used as a measure of quality of the published research items. The assumption behind this approach is that citations are a direct and reliable indicator of the scientific impact of a paper or a journal in its scientific field [1]. Citations count have been used to evaluate the contribution and the scientific performance of individual scientists, research groups, departments, or universities. Hiring and promotion strategies as well as funding and award assignation have been often decided relying on those data [2]. Despite its widespread acceptance, citation-based metrics are not free of flaws. Citations metrics do not consider why an article has been cited, moreover, self-citations practices and to the so-called Matthew effect can alter the citation pattern [3]. Last but not the least, it is the time delay between the article publication, the citations in other published articles, and the indexing in the citations database. In most of the studies about the top-cited articles in dentistry, it is clear how it takes at least a decade for an article to become a citation classic and how older articles are usually cited more frequently, independently of their current impact; while the impact of more recently published papers is usually underestimated due to the short time passed since their publication [4, 5]. Classical citation-based metrics, moreover, fail to describe the impact of research beyond the limits of the academic environment.

Over the last decade, the ways of information interchange among the research stakeholders experienced a major change. With the advent of the so-called Social Web, the scholars discovered many novel options to back-up a published item through the web or by means of social media [2]. All of this online activity around a published item, once invisible, leaves traces. Observing these traces can inform new metrics of scholarly influence and impact the so-called *Altmetrics* or “alternative metrics or alternative metric measures.” The term Altmetrics was first introduced by Priem et al.in 2010 [6].

Altmetrics are measuring how much and what kind of online attention an item is attracting on the web. Altmetrics can measure different types of online activity, such as how often an article has been accessed or downloaded (in form of pdf or HTLM file); uploaded (You tube, Google+); discussed (comments received in journals, scientific blogs, Wikipedia, Twitter, Facebook, or other social networks); bookmarked (e.g., in Mendeley, CiteULike); cited (on sites such as Web of Science, Scopus, CrossRef, Google scholar etc.); or recommended (e.g., on F100Prime).

In the actual scenario, few Altmetrics aggregators are available. An Altmetric aggregator is an online platform that provides an updated overview of the online activity surrounding a published item. The most popular are PlumX and Altmetric.

PlumX is a fee-based web tool from Plum Analytics, a company owned by the Elsevier editorial. PlumX provides five categories of metrics: usage, captures, mentions, social media, and citations. It includes online activities and environments associated with both scholars and laypeople. The analysis of the provided data enables discovery if a published item is being informally and formally discussed and shared in a public or an academic circle [7]. 

**Fig. 1:**
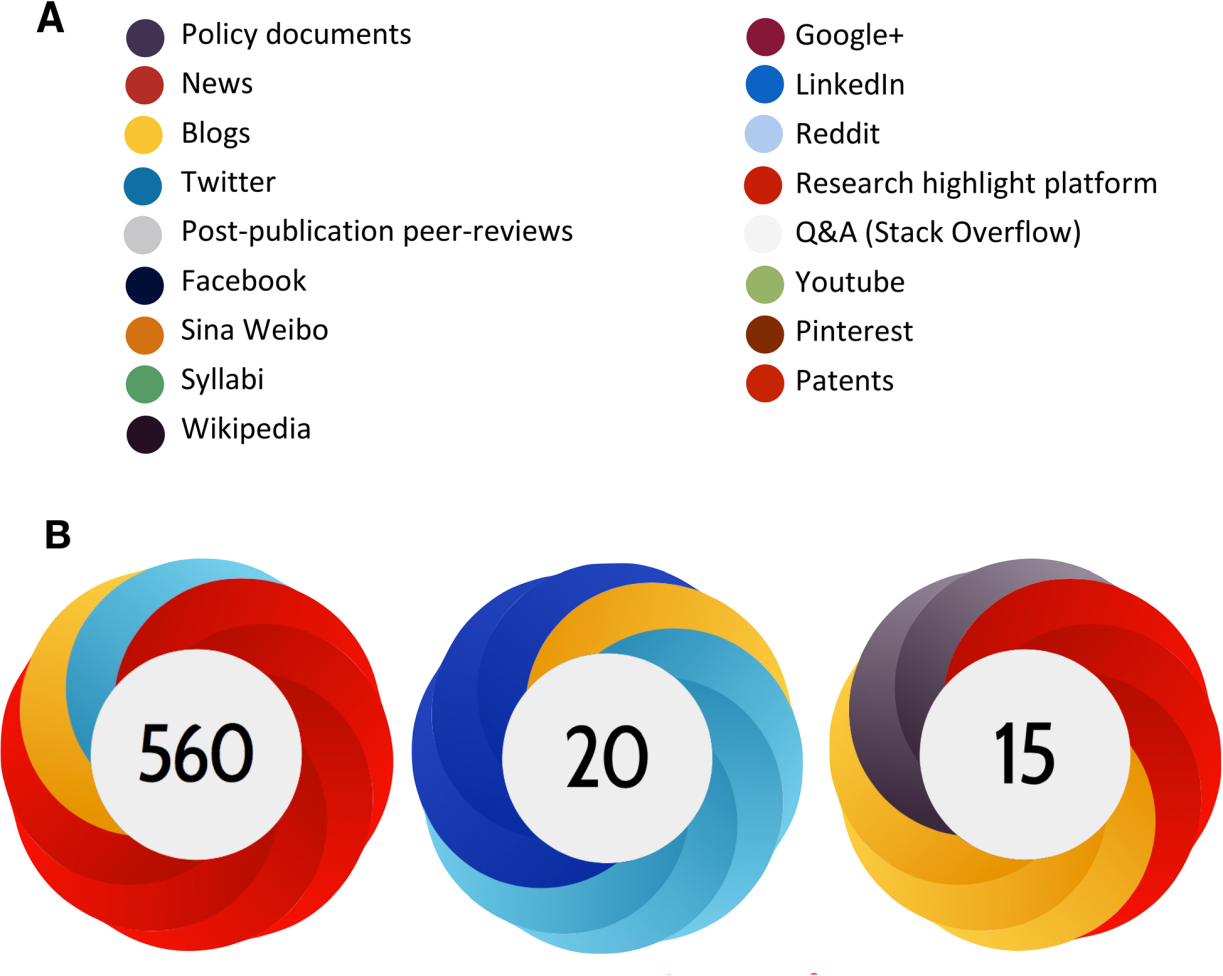
**a** Sources tracked by the Altmetric Explorer. **b** Altmetric donut examples. The prevalence of red colors in the Altmetric donuts indicates that the research outputs received the most online attention from mainstream media, light and dark blue indicates that the item received mainly tweets and Facebook mentions, yellow and grey indicates Wikipedia and blogs activity, while purple is linked to policy documents mentions (starting from the left to the right). AAS is displayed in the center of each badge

Altmetric (Altmetric LLP, London, UK) is a web screening program that aggregates information from three main sources: social media such as Twitter, Facebook, Google+, Pinterest, and blogs; traditional media, both mainstream (New York Times, The Guardian) and science specific (New Scientist, Scientific American), and online reference managers such as Mendeley and CiteULike. Altmetric calculates an Altmetric Attention Score (AAS) for an article based on its mentions in those sources using a specific algorithm [8]. The higher the score, the higher the online attention of the article. Besides the score, Altmetric then generates a visual badge with different colors, and each color represents a different source, offering a visual summary of an article’s impact (Fig. 1).

Altmetric has been adopted by publishers like Wiley and Sons, Springer, and Nature Publishing Group among others. Altmetric, in summary, tracks and analyses the online activity around research outputs, it is a profit service and it provides both a commercial API for customers with all of the application services and a free tool that allows retrieving the AAS and other basic Altmetric data [2]. In the case of Altmetric, the behavior is totally different than in the classic citation system, making it possible for recently published items to acquire a significant recognition and visibility in the short term. The objective of this study was to assess and to analyze the online activity related to research in the field of Orthodontics via Altmetric. Moreover, we wanted to assess if a correlation exists among the Journal Citation Reports (JCR) citations, Scopus citations, Mendeley reader number, and the AAS score.

## Methods

### **Search database**

Firstly, a search was done on the inCites JCR database to identify which journals were included in the JCR during the 5-year period from 2014 to 2018 in the category of *dentistry*, *oral surgery*, and *medicine*. The impact factor of the journals and the quartile rank were registered.

The online activity on orthodontic research was monitored by a search performed by means of the Dimensions Free App (https://app.dimensions.ai/discover/publication) in the Dimensions database.

Dimensions is an innovative online scholarly platform and database, which aims to offer a different perspective on research outputs. Grant awards, journal and book publications, social media mentions, academic citations, clinical trials, and commercial patents are all taken into account to define a research output. The Dimensions Free App only gives access to the publication component that includes items such as articles, chapters, proceedings, monographs, and preprints. The grants, patents, clinical trials, and policy documents components are only accessible with an organizational fee-based subscription and it is a profit service. The citation count is done from the reference list in all publications that have been indexed by Dimensions [9]. Dimensions collects citations from the journals included in the following database: (a) the Norwegian Register for Scientific Journals; (b) The Excellence in Research for Australia (ERA) 2015 journal list; (c) the PubMed indexed journal list; (d) The Directory of Open Access Journals (DOAJ); (e) The Scientific Electronic Library Online (SciELO) journal list; (f) European Reference Index for the Humanities (ERIH) PLUS; (g) the Flemish Academic Bibliography for the Social Sciences and Humanities (VABB-SHW )[10].

The application is able to detect the articles with an AAS higher than one and delivers a break-down of all the Altmetric data involved in the calculation of the aggregated score, comprehensively monitoring the online activity surrounding research articles.

### Search strategy

The search was limited to the nine journals which were listed in the JCR in the year 2018, which were American Journal of Orthodontics & Dentofacial Orthopedics (AJODO), The Angle Orthodontist, The European Journal of Orthodontics (EJO), Orthodontics & Craniofacial Research (OCR), Korean Journal of Orthodontics (KJO), Journal of Orofacial Orthopedics/Fortschritte der Kieferorthopädie, Progress in Orthodontics, the Australian Orthodontic Journal, and Seminars in Orthodontics.

The search was performed by means of the Dimension Free App applying the following filters: publication year (2018 or 2017 or 2016 or 2015 or 2014); source title (American Journal of Orthodontics & Dentofacial Orthopedics OR The Angle Orthodontist OR The European Journal of Orthodontics OR Orthodontics & Craniofacial Research OR Korean Journal of Orthodontics OR Journal of Orofacial Orthopedics/Fortschritte der Kieferorthopädie OR Progress in Orthodontics OR the Australian Orthodontic Journal OR Seminars in Orthodontics).

The search was performed during December 2019 and was limited in time from January 2014 to December 2018. The search retrieved a total of 4301 items that were ordered by Altmetrics Attention Score highest first option.

### Data extraction

The Dimension Free App automatically generated a .csv file displaying the AAS of the selected items. Once downloaded, the data were exported to an excel datasheet (Microsoft Office for Mac 2011 package format).

Three researchers cross-checked the number of retrieved items with the one reported in PubMed and items such as letters, replies, obituaries, table of contents, and adverts were excluded from the final sample that accounted for 3789 published items.

The same researchers screened simultaneously, but not independently, the items with a positive AAS (AAS ≥ 1) and extracted by consensus information regarding the following: (1) article title; (2) journal title; (3) DOI; (4) time interval since publication; (5) Altmetric Attention Score; (6) number of authors and affiliations; (7) type of the affiliation of the corresponding author, i.e., university or other; and (8) country/region of origin as defined by the authors´ institutional affiliations, i.e., USA, Canada, Italy, China.

The different Altmetric data resources contributing to an individual article AAS were recorded after accessing Altmetric through the Dimensions search engine. Additionally, citation counts were harvested from the science citation index expanded from the WOS (property of Clarivariate analytics) and from Scopus (registered trademark of Elsevier BV).

For the articles with an AAS higher than five, the following information was also recorded: (a) article subject, i.e., oral health-related quality of life, (OHRQOL), clear aligners, or others; (b) study type, i.e., systematic review, randomized controlled clinical trials (RCTs), or others.

The study subject categorization was based on the categories proposed by other bibliometric studies published in the field of orthodontics [11]. If the study type was not reported in the title or in the abstract, the full text was analyzed and the study type was identified using the decision tree reported by Grimes and Schulz in 2002 [12].

### Statistical analysis

Descriptive statistics using counts and proportions were used to describe the articles and the journals included in the study. The Pearson’s correlation analysis was used to explore the relationship among citation counts for individual articles, the article AAS, and the total citation count in the analyzed databases. Pearson´s correlation coefficient (*r*) < 0.3 was interpreted as poor, 0.3–0.5 as low, 0.5–0.7 as moderate, 0.7–0.9 as high, and > 0.9 as very high. *p* < 0.05 was considered statistically significant. All statistical analyses were conducted using IBM SPSS Statistics software version 25 (IBM Corp., Armonk, NY, USA).

## Results

As highlighted in Table 1 during the 5 years included in the study, 1085 items out of the 3789 published displayed a positive AAS, and out of these, 128 had an AAS higher than 5. The AJODO gathered the highest cumulative AAS in the studied period but normalizing the AAS according to the number of published articles. The best performing journals were Progress in Orthodontics and the EJO with a mean AAS per published item of 1.351 and 1.455, respectively, while the AJODO reached a 0.82. Progress in Orthodontics was able to attract online attention on the 65% of its published items, while the EJO displayed a positive AAS in 39% of the cases. The AJODO and the EJO published the highest number of items with an AAS higher than 5 but it should be stressed that the number of published articles in the AJODO is a 190.47% higher than in the EJO.

**Table 1:** Number of published items (*N*), total Altmetric AAS, number of citations in WOS and Scopus, mean AAS per article and number of article with a positive AAS (N/AAS), and AAS range for each of the studied journals are presented. (Journal titles are expressed according to their ISO Abbreviation)

Journal			Citations				N/AAS range			
	N	AAS	WOS	Scopus	AAS/article	N/AAS	1	2–5	6–10	> 10
**Am J Orthod Dentofacial Orthop**	*1402*	*1155*	*5648*	*6335*	*0.824*	*427 (30.5%)*	*262*	*121*	*26*	*18*
**Angle Orthod**	*702*	*716*	*3412*	*3805*	*1.020*	*182 (25.9%)*	*110*	*49*	*17*	*6*
**Eur J Orthod**	*481*	*650*	*2446*	*2753*	*1.351*	*186 (38.7%)*	*92*	*52*	*28*	*14*
**Orthod Craniofac Res**	*210*	*155*	*941*	*1078*	*0.738*	*67 (31.9%)*	*39*	*17*	*9*	*2*
**Prog Orthod**	*224*	*326*	*1194*	*1368*	*1.455*	*146 (65.2%)*	*72*	*69*	*1*	*4*
**J Orofac Orthop**	*230*	*41*	*610*	*668*	*0.178*	*28 (12.2%)*	*23*	*4*	*1*	*0*
**Semin Orthod**	*212*	*85*	*301*	*330*	*0.401*	*30 (14.2%)*	*21*	*8*	*0*	*1*
**Korean J Orthod**	*211*	*143*	*878*	*934*	*0.678*	*18 (8.5%)*	*15*	*2*	*0*	*1*
**Aust Orthod J**	*117*	*1*	*179*	*181*	*0.009*	*1 (0.9%)*	*1*	*0*	*0*	*0*
**TOTAL**	*3789*	*3272*	*15609*	*17452*		*1085*	*635*	*322*	*82*	*46*

**Fig. 2:**
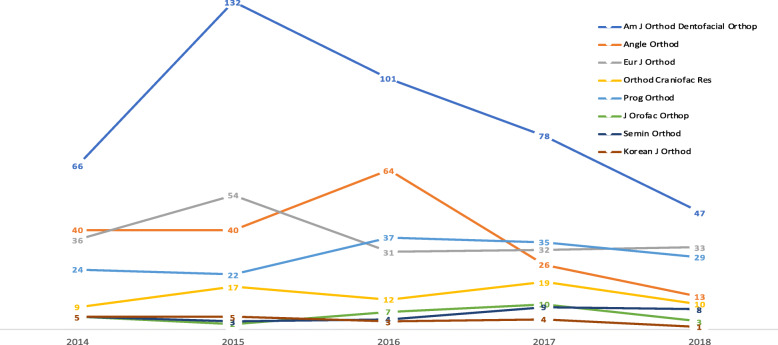
Evolution of the published items with online activity (articles with a positive AAS) from the years 2014 to 2018 in each of the selected journals. The Australian Orthodontic Journal does not appear since only one item displayed a positive AAS in the studied period

According to the data reported in Fig. 2, the online activity of both the AJODO and the Angle presented a decreasing trend during the studied period. Meanwhile, Progress in Orthodontics, the EJO, and OCR presented a quite stable trend of online activity.

The articles with an AAS higher than five were homogeneously distributed along the studied period. Papageorgiou SN., from the University of Zurich, was the most frequent author with 10 published items, being the first author in five of them. The University of London was the most active institution being involved in 14 published items. Fifty-four percent of the authors belonged to a European Institution while only 17.1% belonged to North American ones.

According to the breakdown of Altmetric data, Tweets and Facebook mentions are the most prevalent sources, since Mendeley readers are displayed but not taken into account for the AAS calculation as this type of data cannot be fully audited. Mentions in blogs are the third data source in the AJODO, Angle, EJO, and OCR. Mentions in policy sources are scarce as well as other data source, as highlighted in Table 2. There were no records from Peer review sites, *F1000* reviews, *Reddit* threads, *Q&A* sites, CiteUlike, and book reviews. LinkedIn, Sina Weibo, and Pinterest mentions could not be assessed since they are no longer available because the companies closed their open data stream.

**Table 2:** Breakdown of different Altmetric data resources of the selected journals from 2014 to 2017, and the normalized contribution of each resource is expressed in percentage

	Twitter	Facebook	Mendeley	Google+	Blog	New outlets	Policy source	Wikipedia	Video uploader	Patent	Peer reviews
Am J Orthod Dentofacial Orthop	527	245	14990	12	72	10	0	13	8	3	4
	58.95%	27.40%		1.34%	8.05%	1.12%		1.45%	0.89%	0.34%	0.45%
Angle Orthod	166	89	6435	13	40	30	1	3	3	4	0
	47.56%	25.50%		3.72%	11.46%	8.60%	0.29%	0.86%	0.86%	1.15%	
Eur J Orthod	185	154	6489	10	76	1	2	5	0	0	0
	42.73%	35.57%		2.31%	17.55%	0.23%	0.46%	1.15%			
Orthod Craniofac Res	99	35	1870	0	15	0	0	1	1	4	0
	63.87%	22.58%			9.68%			0.65%	0.65%	2.58%	
Prog Orthod	241	168	5225	12	5	2	0	0	0	0	0
	56.31%	39.25%		2.80%	1.17%	0.47%					
J Orofac Orthop	20	14	222	1	0	1	0	1	1	0	1
	51.28%	35.90%		2.56%		2.56%		2.56%	2.56%		2.56%
Semin Orthod	38	7	880	1	1	4	0	1	0	0	0
	73.08%	13.46%		1.92%	1.92%	7.69%		1.92%			
Korean J Orthod	17	4	476	0	1	14	0	1	3	2	0
	40.48%	9.52%			2.38%	33.33%		2.38%	7.14%	4.76%	
Aust Orthod J	0	1	31	0	0	0	0	0	0	0	0
		100.00%									
TOTAL	1293	717	36618	49	210	62	3	25	16	13	5
	54.03%	29.96%		2.05%	8.78%	2.59%	0.13%	1.04%	0.67%	0.54%	0.21%

When the items are stratified by topic (Table 3), the highest AAS is displayed by items related to OHRQOL. The article with the highest AAS in this category was the one of Lobre et al. in 2016, which investigates the relationship between a micro pulse vibration device and pain perception during orthodontic treatment [13]. This category was followed by “caries and white spot prevention” and “tooth movement acceleration.” The highest AAS per item was displayed by the category injury and complications during treatment due to an article on the harm of carbonated drink to dental enamel [14]. According to the study design stratification, we could assess a very high prevalence of systematic reviews with or without meta-analyses accounting for 67.69% of the articles with the highest online interest, followed by RCTs. The correlation between the AAS and the citations in both WOS and Scopus was poor (*r* = 0.1463 and *r* = 0.1508, *p* < .05). The correlation was poor also with Mendeley reader count (*r* = 0.1115, *p* < .05). The correlation was high between WOS and Scopus citations (*r* = 0.9723, *p* < .05) and was moderate among those citations database and Mendeley (*r* = 0.6879 and *r* = 0.697, *p* < .05).

**Table 3:** Summary statistics for article topic and study design, number (*N*) of articles per topic and study design, Altmetric Attention Score (total), AAS per item and citation count in Web of Science (total), and Scopus (total) are presented

				**Citations**	
**Topic**	**N**	**AAS**	**AAS/item**	**WOS**	**SCOPUS**
OHRQOL	13	335	25.8	213	224
Oral hygiene caries and white spot prevention	12	129	10.8	129	139
Tooth movement acceleration (vibrational, laser, corticotomy)	11	128	11.6	154	171
Class II fixed or removable functional appliances	11	108	9.8	148	167
Stability and relapse/retention/fixed and removable retainers	10	93	9.3	96	99
Eruption problems: impaction, canine ectopic eruption/number problems	7	69	9.9	56	66
Psychological and psychosocial aspects in patients	6	87	14.5	96	110
Periodontics-Orthodontics interaction	6	57	9.5	47	61
Injuries and complications during treatment on both hard and soft tissues	5	197	39.4	9	9
Clear aligners	4	54	13.5	98	97
Brackets design, friction, self-ligating	4	49	12.3	65	58
Habit and myfunctional problems influence on orofacial structures	4	44	11.0	21	17
CBCT/digital model/3D Technology	4	39	9.8	58	67
Early/interceptive treatment	4	32	8.0	29	29
Bone anchor	4	32	8.0	27	30
Archwires, elastomers, resins and other materials: effectiveness, biochemistry, biology, toxicity	3	54	18.0	8	11
Orthopaedic treatment of Skeletal Class III	3	35	11.7	34	29
Aesthetic soft tissues—profile evaluation, smile evaluation	3	27	9.0	27	30
Sleep disorders/breathing	2	31	15.5	7	8
Artificial intelligence application to orthodontic treatment and diagnosis	2	21	10.5	10	11
Vertical alterations: open bite	2	21	10.5	8	11
Others	8	83	10.4	107	117
				**Citations**	
**Study design**	**N**	**AAS**	**AAS/item**	**WOS**	**SCOPUS**
RCTs	32	521	16.3	344	359
Systematic review	30	340	11.3	480	535
Observational (cross-sectional, longitudinal, cohort)	28	311	11.1	211	224
Systematic review and meta-analysis	25	282	11.3	294	324
CCTs	5	61	12.2	42	65
Meta-epidemiological study	2	23	11.5	19	24
Case series/case report	2	14	7.0	18	18
In vitro	1	122	122.0	1	1
Editorial	1	41	41.0	3	2
Bibliometric	1	13	13.0	3	2
Scoping review	1	8	8.0	2	1

## Discussion

Despite the availability of two web tools, such as PlumX and Altmetric, we decided to use the latter for our investigation. Firstly, because despite Altmetric being a pay-per-use service, the Altmetric data are freely available through the Dimensions Free App, while PlumX data cannot be accessed freely. Moreover, most of the Altmetrics studied in medicine and dentistry relied on Altmetric data allowing a better discussion and analysis of the results. According to many authors [15, 16], Altmetric represents the most comprehensive and updated source of social media and mainstream media data related to scientific articles. As a future development of our work, it would be interesting to access PlumX data and assess if the information gathered from the two tools is in accordance.

To our best knowledge, this is the second study to report on Altmetric in Orthodontics. Livas and Delli in 2017 analyzed the 200 articles in the Orthodontic literature which received the highest AAS [15]. We did not want to limit our analysis to the most cited articles because we wanted to give a broader insight into the impact of this new metric in Orthodontics. In the 5 years included in the study, only 28.63% of the articles received a positive AAS, a percentage that is lower than the 34.62% reported by Kolahi et al. in 2017, who analyzed the entire dental literature of 2015 [17]. Comparing the result of Kolahi et al. with ours, the difference in time between the two reports should be considered, given the extreme speed of web data flow. According to what was reported in implant dentistry literature, the AAS displayed a nine-fold increase comparing the top-cited journals in 2013 and 2016 [18]. A difference of as little as 1 year could result in a tremendous change in AAS value. The percentage of articles with a positive AAS presents a huge variability among the journals, being 65% in Progress in Orthodontics and just 0.9% in the Australian Orthodontic Journal. The same huge variability was observed in a sample of pediatric dentistry journal and periodontics journals [10, 19]. The variability could be explained by the different online activity among the studied journals.

Progress in orthodontic, the journal that displays the better behavior in terms of AAS, offers Altmetric data of every published article but have not embedded the Altmetric badge to its website, while the EJO and OCR have already included the Altmetric badge to their articles and websites. Ortega in 2017 in an investigation about Twitter accounts and AAS, suggested that the presence and the activity of a journal or of the journal editor on social media is a key factor in contributing to the social dissemination of the journal production [20]. A key role may be played also by the topic of the published articles since, as reported by Holmberg and Vainio in 2018, depending on the platform, the personal connection to the research topic, emotionally appealing subjects, and the novelty of the research topic are often mentioned by the authors as reasons for the received online attention [21]. 

In our data, Twitter activity represents the most frequent Altmetric data resource in all of the journals, while Facebook was the second one (Table 2). The global ratio between tweets and mentions on Facebook walls was 1293/717 in our study group, while it was 11510/1967 in the whole dental literature in 2015, 466/59 in pediatric dentistry journals, and just 1871/193 in periodontics journals [10, 19, 22]. In 2018, Kolahi et al. reported that tweets are the main Altmetric data resource that determine the global AAS, while Facebook activity represented only 3.76% of the total activity in dental literature in 2015, having the lowest relative importance when compared with the other analyzed items [22]. Those data could point out how orthodontists are more used to sharing their research output on Facebook when compared to colleagues in other branches of dentistry.

Since the AAS is mainly derived by Twitter and Facebook posts, there is a major concern about data manipulation that can be considered fairly easier than the one involving the classical citation counts. Social media is easily accessible, and in the web, it is operating many spam companies by selling votes or tweets from registered users. Altmetric claims to use a specific algorithm to limit the effect of such misconducts by tracking down artificial patterns of attention or detecting social media automation tools [10]. 

The very low number of patents in the studied period could indicate a sort of disconnection between orthodontic research and the industry in contrast to the findings in periodontics, endodontics, and implant dentistry [10, 18, 23].

We decided to limit the topic and study design analysis to the items with an AAS higher than five according to what suggested by Kolahi [23] in order to allow a more rigorous sample size determination. Since with a single self-tweet it is possible to gather an AAS of one, to extend a more detailed analyses to these articles could have led to an important distortion of the results.

When the published items were stratified by study subject (Table 3), OHRQOL received the highest AAS score according to what was previously reported in pediatric dentistry [19]. Livas and Delli, in a previous Altmetric study in Orthodontics, reported similar results, being OHRQOL one of the topics that displayed a higher AAS after socio-demographic studies and research on new technologies [15].

The second article for AAS score in our sample was an article on erosion, one of the trending topics highlighted previously in paediatric dentistry [19].

The highest AAS were reached by articles related to news outlets, which have a higher relative weight in the overall score calculation. The article of Lobre et al. was mentioned in 25 articles in online and traditional press that focused on the ability of Acceledent® to decrease the discomfort related to the orthodontic therapy [13]. Also according to Livas and Delli in 2018, this article displayed the highest AAS on the 200 most cited articles in orthodontics [13] and how even after 2 years since the publication of the article the interest keeps high on this topic. The article of Ryu et al. on erosion was mentioned in 16 press articles focusing on the drawback of carbonated drinks and how refraining from these drinks can allow people to live better and longer [14]. In both cases, only part of the information was extracted from the article and was used to attract the interest of the reader. The behavior of the article of Pithon et al. in 2014 on dental aesthetics influence on finding a job was different, since its AAS was mainly due to tweets and Facebook mentions [24].

There can be therefore several factors behind a high AAS. In some cases, the article focuses on a subject that is appealing to the general public and can attract the media’s attention. In other cases, the topic can be sensitive for academics, patients, or laypersons involved or interested in a specific treatment or pathology and is disseminated in social media. Both social and classic media focus on laypeople, with a limited knowledge of medical topics, and different interests and sensitivity from the academic users that are the usual target of traditional metrics. Altmetric results interpretation must be done carefully due to the early stage of this metric.

The online activity of research can provide a different, broader, and updated picture of an article’s impact. Altmetric data seem to be able to measure the broader impact of research, including the social impact, which in this case is the social improvements obtained from the transference of research results, and according to some authors can therefore play a major role in its assessment [25]. 

Delivering access to orthodontic research to the general public can increase the social impact of our discipline and make the public aware of the benefits of the treatment on the global wellbeing and quality of life of an individual as well as the negative effects of a do-it-yourself approach that represent a present and a future threat of our profession. Due to the tremendous potential of dissemination at a global level of social media, it is pivotal to ensure that any knowledge transfer and public engagement is based on scientific rigor in order to maintain the credibility of our profession.

The stratification of data by study design (Table 3) displays a high prevalence of systematic reviews with or without meta-analysis and RCTs. In Table 3, the published item editorial was included, despite not being considered a study design, because it was able to attract considerable online attention and our aim was to identify every published item able to get a significant AAS. The results are similar to the ones reported in Altmetric studies performed in cardiovascular research, in Neurointervention, Endodontics, and Periodontics [10, 23, 26, 27].

Both systematic reviews and RCTs rest at the top of the evidence pyramid. It is indeed surprising how these high-quality articles are rarely used for evidence-based policy making as highlighted in the Altmetric data break-down. On the contrary, many studies performed only on citations count report that the most cited items are expert opinions and case-series with a low level of evidence [5]. Altmetric is not intended to be a measure of scientific evidence, and this correlation can be due to its immediacy. Altmetric is able to highlight newly released research items, among which, systematic reviews and RCT have a higher frequency than among the most cited items which are usually at least one or two decades old [5, 28].

In our sample, the correlation between the citation count in the WOS and Scopus with the AAS was poor along the studied period. This result is in agreement with other authors in different fields of dentistry, who observed no correlation between the AAS and the citations count in Scopus or WOS [10, 15, 19, 23]. In the previous investigation in Orthodontics by Livas et al., no correlation was observed between AAS and citation in Scopus (*r* = 0,09; *p* = 42), while the correlation in WOS was not assessed by these authors [15]. In other fields of medicine, some authors found a moderate correlation [19]. Huang et al., in 2018, concluded that in their study sample of 2406 articles published in Plos medical journals, the ones with the highest AAS have a higher chance of displaying a higher number of citations [2]. Disregarding their possible correlation, AAS and citation count are measuring different aspects of a published item, since the AAS measures online diffusion and citations measure how the researchers are using a previous research output to set up, back up, or discuss their own research. It takes much less time to discuss a publication on social media than to acquire new citations. Citation-based assessment of an article is not a fast process, and a long time is often necessary before the value of an article is completely recognized. On the contrary, Altmetric data usually appear quite quickly. As it occurs for citations, the AAS is also field sensitive, and some authors argued they cannot be compared without a time and field normalization of the raw data [29]. Further research is needed to clarify if and in which aspect a correlation exists between those indicators.

Finally, we found a moderate correlation between Mendeley readers count and citations in our sample, both in WOS and Scopus. Our findings are in agreement with the ones of other authors [23, 30, 31]. This finding is interesting, and if validated by future research, Mendeley reader count could be used to detect in advance the area of potential research interest or the top-ranked articles, being the Mendeley reader count much faster than the classic citation approach.

Our study presents some limitations in the literature search since we only analyzed the articles published in the orthodontic journals listed in the JCR and we did not include all those publications related to orthodontics which were published in non-specialty journals and those publications published in specialty journals not listed in the JCR such as the Journal of Orthodontics or the Journal of Clinical Orthodontics. The inclusion of these journals could have provided a broader insight into the impact of this new metric in the orthodontic field. The orthodontic specialty journals are more prone to publish highly focused and specialized papers, for example, comparison of two different cements and loss of strength of elastomers over time,, which are relevant for orthodontic clinicians and researchers, but may not be interesting for the general public and therefore disseminated via social media. Non-specialty journals, such as dental journals, may include articles of broader interest to the public, as they are not geared specifically toward orthodontists. The methodology of our search may therefore underestimate the online attention of orthodontic research. As a future development, we could consider if a study involving non orthodontic journal leads to different conclusions. Moreover, the correlation of the citation count in the WOS and Scopus was only performed on the article with a positive AAS and was not expanded to all the other articles. This aspect will need further investigation.

## Conclusions

Within the limitations of the present study, online attention to orthodontic research can be improved. Progress in Orthodontics and the European Journal of Orthodontics displayed the highest level of web activity. OHRQL studies appear to have significantly higher visibility on social and traditional media. Altmetric combined with traditional metrics could get a broader insight into research effects. No correlation exists between the citation count in WOS and Scopus and the AAS, while the Mendeley user count has a moderate correlation with citations. In order to facilitate the dissemination of the research output in non-scholar audiences, journal-related social media accounts should be implemented. When reporting the conclusions of their research, the authors should report not only the practical or clinical implications but also the impact of the findings on the general public in an easy to understand way, pointing out the limitations of the results in order to avoid an opportunistic use of the research outputs.

## Data Availability

All the relevant datasets used and/or analyzed during the current study are within the paper, and any additional dataset is available from the corresponding author on reasonable request.

## Abbreviations

AAS: Altmetric Attention Score;
AJODO: American Journal of Orthodontics & Dentofacial Orthopedics
EJO: European Journal of Orthodontics
API: Application
JCR: Journal Citation Reports
KJO: Korean Journal of Orthodontics
OCR: Orthodontics & Craniofacial Research
OHRQOL: Oral Health-Related Quality of Life
RCTs: Randomized controlled clinical trials
WOS: Web of Science
